# Supplementary material for: Deprescribing Anticholinergics to Preserve Brain Health: Reducing the Risk of Dementia through Deprescribing (R2D2): Study Protocol for a Randomized Clinical Trial
Source: Res Sq. 2024 Oct 28:rs.3.rs-4682599. Preprint. [Version 1] doi: 10.21203/rs.3.rs-4682599/v1 (PMC11581051; doi:10.21203/rs.3.rs-4682599/v1)
Supplement: Supplement 1 [file NIHPPRS4682599V1-supplement-1.pdf]

Supplementary Files

This is a list of supplementary files associated with this preprint. Click to download.

- R2D2InterventionProceduresFinal6.2024.docx
- R2D2ProtocolGeneralDeprescribingRecsFINAL.docx
- R2D2SPIRITchecklist7.29.24.docx
